# Typhoid Fever in Country Practice

**Published:** 1901-09

**Authors:** J. A. Knight

**Affiliations:** Eatonton, Ga.


### TYPHOID FEVER IN COUNTRY PRACTICE.

BY J. A. KNIGHT, M.D.,
EATONTON, GA.

As a great deal has been written in your valuable journal during the past year, by the best talent of the medical profession in Georgia, upon typhoid fever and its treatment, allow me to say as a preface to these few remarks, that I have no more interest in Mr. Merrell than any other manufacturing chemist of America, no more interest in his solution of bismuth and hydrastis, than in the old time honored turpentine emulsion, carbolic acid, iodine, or the more modern methods of Woodbridge, Rice, Hubbard and others, based upon scientific principals, elimination and antiseptics.

I will also add, that any physician who will make claims to any treatment, not based upon scientific principals, or actual experience, is worse than the man of old, who sold his birthright. A traitor to humanity, and to the interest of his profession.

It is not my purpose to write an article on typhoid fever, or discuss its etiology, or much of its morbid anatomy. From time immemorial it has been written from every standpoint, by the best element of the profession, and a legion of remedies advised, many of which have perished, unwept and unsung, and but few have gained much notoriety or achieved their object, as it claims annually for its victims thousands of the pride of earth. Like the poor, we have it with us all the time; and I have treated it upon every principal advised by the best authorities from turpentine emulsion of Wood, method of Brandt, Woodbridge, Rice and others too numerous to mention. I have nothing to say against them, and while my fondest hopes have been realized in the action of Merrell's solution, unlike Ephraim of old, I am not joined to my idols, and in that spirit of fairness and progress that has always characterized me, am ever ready to employ any rational method which offers improvement.

As to the morbid anatomy of typhiod fever, we find a catarrhal condition throughout the small and large intestine. This fact is based upon thousands of antopsies.

It frequently begins as a localized catarrh of the stomach and from errors in treatment, or diet, it extends rapidly over the mucous membranes, and the violence and duration depend upon the amount of tissue involved and complication which subsequently develop.

To this catarrhal condition (according to the views of Osler and other gentlemen of prominence) is due the diarrhea and the great source of danger, and to this condition I direct my treatment, whether we accept the bacilli theory of Eberth, Graffky and Koch, or regard it as a pestilence that lurketh in the darkness.

The indications for treatment are too plain for any physician who understands the effect of bismuth and hydrastis upon an inflamed mucous membrane, not to appreciate its value and *modus operandi.* It is absolutely unirritating and pleasant to take. Its action is prompt, and after moving bowels well with calomel and soda (five grains each) every four hours, I saturate contents of stomach with the solution, forty drops every four hours. I insist that many of the complications we meet are due to errors in treatment and diet. I admit this is true of my own experience, and we should prescribe and feed with judgment and discrimination. If tympanites or constipation should arise we should investigate the cause, and give Marchand's glycozone in ʒi doses three or four times a day, as conditions may suggest. It is a powerful germicide, and while not a cathartic *per se*, it has a favorable influence upon the stomach, produces mild peristalsis without unpleasant sequelæ or pain—hence no danger of perforation.

Quinine given for even diagnostic purposes has, in my hands, proven a dangerous experiment. It disturbs the stomach, demoralizes the digestion, increases the intensity of the catarrh alluded to above, and its continued use is a source of danger. The morning remission so common to the early stages of typhoid fever offers a great temptation to give quinine.

When a trial is thought to be due the patient, give it early and in large doses, that with one fell swoop, as it were, we are made to realize the mistake early and remedy the evil before the patient's vitality is exhausted. Insomnia, if present, should receive prompt attention. The low, muttering delirium is frequently mistaken by the nurses for sleep. If allowed to continue, you shall soon find your patient with violent mania, which is very annoying.

I am opposed to opiates upon general principles. They disturb the stomach, arrest the secretions, oppose elimination, and favor autointoxication. When complications suggest it, I have given heroine hypperdermically, one twenty-fourth grain, with fair results; but it should be remembered that it is a morphine derivitave, and should be used with care.

Trional is in many cases objectionable. Bromidia has always given me good results. I sometimes combine it with papine, ʒi each at bed time, which secures for patients several hours' sleep.

Spinal irritation and a condition of hyperemia about the base of brain is another complication that demands attention. I treat it with small applications of cantharidal collodion behind the ears and over the spine, as points of tenderness may suggest. This may be apractice of barbarism, but I find it almost indispensable.

Cystitis in my section is a frequent complication, and very annoying to the patient. I have known it to continue hours, and even days. I have found two or three cystogen tablets quite sufficient to relieve it. It is comparatively a new preparation to me, and I cannot recall its formula; but the evolution of formaldehyde in urine is the *modus operandi*. Diarrhea is a condition that I no longer fear since using the solution of bismuth.

With my old methods of treating fever I found it in almost every case, and it was a great source of danger.

Right here allow me to state that since my first communication upon this subject, three years ago, I have received more than one hundred letters of congratulation and inquiries from every section of the United States in reference to its action. Among them a letter from Dr. B. A. Check, Marion, N. C., saying he received the article just in time to save a valuable life in his town, and better still it was a lady's life, which has more than repaid me for all the trouble and study connected with this subject. Pardon this digression.

DIET.

One of the greatest mistakes of my life has been a routine habit of allowing sweet milk *ad libitum*, and I have witnessed the most fearful consequences due to its use. I removed last year a man from a hospital not a great distance away, who was dangerously ill with typhoid fever. His treatment for several days consisted of 60 grains of quinine daily and about three pints of sweet milk. Notwithstanding no microscopical tests had been made for the malarial plasmode, his doctors supposed it to exist from the fact that he had lived in a malarial section. He was with me one week before he realized or knew he had been removed to my home. I never witnessed such tympanitis and delirium; but as soon as I relieved him of his sweet milk and *Quinia diabolica* and placed him upon appropriate treatment he made a good recovery. I can enumerate many similar cases. Very little feeding is necessary during the early stages of typhoid fever if you have a fairly good subject to begin with.

BUTTERMILK.

Horlick's malted milk and liquid peptonoids given as indicated meet all the requirements. I have no figures, or statistics, by which I can give the average duration of cases thus treated, but I abort it, or allow it to abort itself, any where from ten to twenty days. I repeat that I have nothing to say in condemnation of any rational treatment of typhoid fever.

Theoretically, some of them do not seem to meet the indications.

"But all's well that ends well."

I have seen many cases of typhoid fever in which the stomach could not tolerate either menthol or guiacol. I have known many cases of fever, simple and complicated, to run a period of five or six weeks with Woodbridge treatment faithfully and persistently employed from the initial symptoms.

We have no systemic antiseptics, and consequently I cannot indorse viskolein solution, and so informed the company. They are gentlemen of integrity, and manufacture some of the finest products on earth.

I was taught to treat typhoid fever with turpentine, internally, externally and eternally, and for years I carried it out faithfully, and found many objections to its use. In casting around for a better remedy, my attention was called to carbolic acid and iodine.

Theoretically, it filled the bill; but I found that many stomachs would rebel, then came the mineral acids, which apart from their tonic properties, the results obtained were absolutely negative. Along these lines of treatment I met with average success. Diarrhea was present in almost every case, hemorrhages not uncommon, and death rate ten or twelve per cent. among the best specimens of the races; but even this was an improvement from the days of old lang syne, when the mortality in my county was fearful, and the average duration was too long to calculate. I have met with old men all through my professional career who tell me of an attack of typhoid fever once upon a time which held them down three or four months, and they positively assert had they not received the very best nursing and medical attention, they long since would have dwelt with the angels. Surely this a day of progress.

A knowledge of the germ theory has completely revolutionized the practice of medicine, or evolution has changed the character of diseases.

